# Reproducing during Heat Waves: Influence of Juvenile and Adult Environment on Fecundity of a Pest Mite and Its Predator

**DOI:** 10.3390/biology12040554

**Published:** 2023-04-05

**Authors:** Thomas Tscholl, Gösta Nachman, Bernhard Spangl, Hanna Charlotte Serve, Andreas Walzer

**Affiliations:** 1 University of Natural Resources and Life Sciences, Vienna, Department of Crop Sciences, Institute of Plant Protection, Gregor-Mendel-Straße 33, 1180 Vienna, Austria; 2 University of Copenhagen, Department of Biology, Universitetsparken 15, DK-2100 Copenhagen Ø, Denmark; 3 University of Natural Resources and Life Sciences, Vienna, Department of Landscape, Spatial and Infrastructure Sciences, Institute of Statistics, Peter-Jordan-Straße 82/I, 1190 Vienna, Austria

**Keywords:** Phytoseiidae, Tetranychidae, biological control, climate change, heat stress, predator–prey interactions, reproduction, non-consumptive predator effects

## Abstract

**Simple Summary:**

Heat waves experienced early in the lives of arthropods can affect their sensitivity to heat stress as adults. Thus, juvenile acclimation may influence fine-tuned predator–prey relationships since the two opponents respond differently to heat stress. Here, we exposed heat wave-acclimated and non-acclimated females of the predatory mite *Phytoseiulus persimilis* and its prey, the spider mite *Tetranychus urticae*, to extreme and mild heat waves and assessed their reproductive performance on bean leaves. Additionally, ovipositing prey was exposed to predator cues during heat waves. Our results showed that juvenile acclimation decreased the tendency of both species to escape from the leaves, but younger and more fecund predator females left the leaves than prey females under extreme heat waves. Reproduction in both species increased under extreme heat waves but was not affected by juvenile acclimation. Additionally, predator cues lowered the oviposition rates of prey, but the effect was marginal compared to the strong positive effects of heat waves. Our results indicate that control of spider mites by *P. persimilis* may become less efficient under extreme heat waves, partly because the predators suffer more than their prey from high temperatures and partly because escaping predators risk dying before they find a suitable place to feed and oviposit.

**Abstract:**

The thermal history of arthropod predators and their prey may affect their reproductive performance during heat waves. Thus, a matching juvenile and adult environment should be beneficial as it enables the individuals to acclimate to extreme conditions. Prey fecundity, however, is also affected by a second stressor, namely predation risk. Here, we assessed the impact of extreme and mild heat waves on the reproductive output of acclimated (juvenile and adult heat wave conditions are matching) and non-acclimated females of the biocontrol agent *Phytoseiulus persimilis*, a predatory mite, and its herbivorous prey, the two-spotted spider mite *Tetranychus urticae,* on bean leaves. Their escape and oviposition rates and egg sizes were recorded over 10 days. Additionally, ovipositing prey females were exposed to predator cues and heat waves. Acclimation changed the escape rates and egg sizes of both species, whereas fecundity was only influenced by the adult thermal environment via increased egg numbers under extreme heat waves. Acclimation reduced predator and prey escape rates, which were higher for the predator. Pooled over acclimation, both species deposited more but smaller eggs under extreme heat waves. Acclimation dampened this effect in prey eggs, whereas acclimation resulted in smaller female eggs of the predator. Prey deposited larger male and female eggs. Predator cues reduced prey oviposition, but the effect was small compared to the large increase gained under extreme heat waves. We argue that the success of predators in controlling spider mites during heat waves mainly depends on the fates of escaping predators. A permanent absence of predators may result in the numerical dominance of prey.

## 1. Introduction

The biotic and abiotic environments of an organism are usually subject to changes such as those in weather conditions, predation risk, and food availability. Therefore, the capability to cope with changing environments is vital for all organisms. An important aspect influencing the possibility of adapting to these changes is the factor of time: how fast and often do environmental changes occur and how long do they last? Changes over longer time scales, such as the slow increase in global mean temperatures, may allow individuals to adjust based on genetic adaptations [1]. In contrast, the occurrence of unpredictable heat waves with a relatively short duration causes rapid thermal changes, which require fast responses from the exposed individuals. Such conditions may favor phenotypic plasticity [2] and result in heat wave-acclimated phenotypes [3].

Based on empirical [4,5,6,7,8] and mathematical studies [9], the sensitivity of species to heat waves is often dependent on their trophic classification: higher trophic levels (e.g., predators) are less heat-resistant than lower trophic levels (e.g., prey). Heat wave-induced destabilization of the finely tuned relationships and dramatic changes in the population dynamics in favor of the prey might be the consequences of predator–prey interactions [6]. The causes for these phenomena remain ambiguous; owing to their higher mobility, the predators may be better able than their prey to evade heat stress by moving away. On the other hand, if the predators are unable to escape, they may suffer more than their prey with respect to feeding, reproduction, survival, and development. Consequently, heat waves are likely to cause a decline in the predator-to-prey ratio [10,11].

Fecundity is a crucial component of individual fitness for arthropod predators and prey, enabling their population growth and persistence [12]. This heat-sensitive trait [13] might be influenced differently depending on whether a female has already experienced thermal stress during juvenile development or only as an adult. In the former case, high temperatures often induce irreversible shifts in pivotal traits such as age and size at maturity [14]. These changes may also alter the morphological, physiological, and behavioral traits of adult females through juvenile acclimation [15], thereby affecting their reproductive performance [16,17,18,19]. These shifts are considered adaptive when the juvenile and adult thermal environments match (e.g., [20,21]). Alternatively, only the thermal environment during adulthood may influence reproduction because developmental canalization might be capable of buffering juvenile traits against thermal perturbations [22,23]. However, a general trend in how the environment experienced by a female during the juvenile and adult stages affects her subsequent fecundity cannot be deduced from the literature (e.g., [15,16,17,18,19]), indicating highly species- and trait-dependent implications on reproduction. Additionally, heat wave stress on reproduction of prey may interact with another stressor, namely predation risk [24]. Separate heat wave and predation risk effects provide contrasting results on the reproduction of prey. When the food resource is not a limiting factor, higher temperatures commonly increase fecundity until the species-specific thermal fertility limit is reached [12]. In contrast, the presence of predators usually reduces the reproductive output of prey [25,26]. However, the combined heat wave and predation risk effects on prey fecundity are rarely evaluated in terrestrial predator–prey interactions.

Hence, here we assessed the effects of heat waves on the reproductive output of the herbivorous pest mite species *Tetranychus urticae* Koch (Acari: Tetranychidae) and its common biocontrol agent, the predatory mite *Phytoseiulus persimilis* Athias-Henriot (Acari: *Phytoseiidae*). *Tetranychus urticae* is a polyphagous pest able to build characteristic webs covering its food plants and host high population densities. The use of *P. persimilis* became an effective alternative to the use of chemical pesticides since this predator feeds on all developmental stages of *T. urticae*, thereby achieving high control efficacies [27]. Both species are characterized by high developmental rates, thereby reaching adulthood in a few days [7,8]. The reproductive output of both species is highly dependent on the availability of food resources for the females, classifying them as income breeders. *Tetranychus urticae* reproduces via male-producing parthenogenesis (arrhenotoky), since unfertilized eggs become males and fertilized eggs become females [28]. *Phytoseiulus persimilis* uses pseudo-arrhenotoky, where males also develop from fertilized eggs, but the paternal set of chromosomes is eliminated during early embryonic development [29]. Under favorable conditions, the sex ratio of populations of both species is female-biased (3:1—females:males), but can vary depending on many intrinsic and extrinsic factors [30,31]. In both species, male eggs are smaller than female eggs based on their mean sizes, but their overlapping size ranges do not allow for an exact separation between male and female eggs [32]. Usually, a single mating is enough to secure life-long fertilization in both *T. urticae* and *P. persimilis* females [33,34]. On average, *T. urticae* females lay about 160 eggs during their lifetime [35], while the corresponding value for *P. persimilis* is about 80 eggs [34].

There is the tendency that the fecundity of the predator is more negatively affected by dry, hot climatic conditions compared to the prey [36,37,38,39,40,41,42,43]. However, most studies used constant temperatures and did not include humidity effects (but see [44]). Additionally, the consequences of juvenile heat wave acclimation on female fecundity have not yet been investigated in our predator–prey couple, although heat waves led to changes in the age and size at maturity of both species [7,8]. Under extreme heat waves, the species developed faster compared to mild heat waves. This may lead to an earlier start of mating and reproduction, resulting in a higher capacity for population increase [30]. In contrast, the effects on size at maturity are species-specific: female predatory mites became smaller when developing during extreme heat waves, while female spider mites were able to maintain their size. These changes may favor the prey, since large females are usually more fertile [45,46,47].

Moreover, the reproductive output of the prey may also be affected by encounters with hunting *P. persimilis*. Since this predator species only attacks and kills spider mites of the genus *Tetranychus*, it is highly adapted to locate spider mite prey on plants [48]. Thus, when prey and predator are present on the same plant, prey females are often confronted with chemosensory volatile cues and/or contact cues induced by the predator but acting as kairomones for prey. Volatile cues are perceived by spider mites from a distance and signal that predators are likely to occur in the surroundings. Contact cues are perceived by touching them, indicating that at least one predatory individual is, or has recently been, on the same leaf as that currently occupied by spider mites [49]. Consequently, volatile cues may constitute a low predation risk, while contact cues represent an acute and, therefore, a high predation risk [50]. Prey females are able to discriminate between the two types of cues [25], which may allow behavioral adjustments according to the degree of predation risk. However, predation risk may interfere with heat wave effects, leading to other oviposition decisions in prey females than when they are exposed to only predation risk.

Since heat waves are expected to increase in both frequency and intensity [51], this study addresses how heat waves affect the reproduction of these two interacting species. In particular, we expect that: (1) mobile predators are more heat sensitive than the prey, resulting in higher escape rates, whereas the less mobile prey suffers more from mortality; (2) the reproductive output increases under extreme heat waves in both species at the expense of smaller offspring egg sizes; (3) juvenile thermal acclimation (i.e., matching of juvenile and adult heat wave conditions) has benefits but also costs for both predator and prey females; and (4) confronted with predation risk, the prey is able to adjust its anti-predator behavior in a threat-sensitive manner, but predation risk effects on reproduction are low relative to heat wave effects. Therefore, we conducted a series of laboratory experiments by exposing acclimated and non-acclimated adult females of both species to extreme and mild heat waves to assess their escape behavior and reproductive performance (adult survival, food consumption, egg numbers, egg sizes, and sex ratios). Additionally, the responses of spider mites to low predation risk (volatile cues) and high predation risk (contact cues) were assessed by measuring their consumption and fecundity rates under mild and extreme heat wave conditions.

## 2. Materials and Methods

### 2.1. Mite Origin and Rearing

Specimens of *P. persimilis* and *T. urticae,* originating from a commercial producer (Biohelp, Vienna, Austria), were used to establish lab populations, which were reared in an incubator [25 ± 1 °C, 60 ± 10% RH, 16:8 (L:D) h] and in a climate chamber [25 ± 2 °C, 60 ± 15% RH, 16:8 (L:D) h], respectively. These climatic values are optimal to rear the mites and correspond to the standard in labs dealing with these species [7,8,38,44]. Whole bean plants (*Phaseolus vulgaris* L.) served as host plants and habitat for the stock population of *T. urticae*. Arenas, consisting of acrylic tiles on foam cubes in acrylic boxes (20 × 20 × 6 cm) half-filled with water, were used as rearing units for the predator populations. Bean leaves infested with spider mites were added three times per week.

### 2.2. Mimicking Mild and Extreme Heat Waves

We analyzed the frequency, intensity, and duration of heat waves in Eastern Austria from May through September in the years 2011–2020 based on the daily measurements of minimum (T_min_) and maximum (T_max_) temperatures. Data were obtained from a meteorological station located in Eisenstadt (48.117° N, 16.567° E). The station belonged to the Central Institute for Meteorology and Geodynamics (ZAMG), and the data are considered representative of Eastern Austria. Based on the definition of heat waves in Central Europe [52,53], we evaluated the characteristics of the heat waves in this decade. The mean duration of the 28 heat waves was 9.9 days (3–29 days). The daily T_min_ and T_max_ values ranged from 13.3 to 24.3 °C (mean 18.8 °C) and 30.1 to 39.3 °C (mean 31.5 °C), respectively. Daily T_max_ values of ≥35.0 °C were only recorded for 26 days (14.05%), whereas the daily T_max_ was below 35.0 °C for the majority of heat wave days (159 days, 85.95%). Based on these thermal data, we generated daily climate patterns that mimicked mild (T_max_ = 32.0 °C) and extreme (T_max_ = 38.0 °C) heat waves (Table 1). Because of their high frequency, mild heat waves are considered to be representative of present climate change. In contrast, extreme heat waves are representative of future climate change since the daily T_max_ should correlate linearly to the global mean temperatures.

### 2.3. Female Rearing Units, Starvation Cages, and Experimental and Sex-Determination Units

To obtain similarly aged and fertile females for the experiments, 15–20 females of either *P. persimilis* or *T. urticae* were taken from the rearing units and placed on detached bean leaves. The predators were also provided with spider mites. After 6 h, the females were removed so that the deposited eggs were ±3 h old, which should minimize the confounding effects of the maternal thermal environment [54]. These rearing units were then either exposed to heat wave conditions (mild, extreme) or to constant climatic conditions [25 ± 2 °C, 60 ± 15% RH, 16:8 (L:D) h] during juvenile development in order to obtain heat wave-acclimated or non-acclimated females. After reaching adulthood, the young females (3 to 4 days old) were placed singly in closable acrylic starvation cages with circular chambers (Ø 15 mm, 3 mm high) for 24 h and exposed to constant climatic conditions of 25 ± 1 °C, 60 ± 10% RH, and 16:8 (L:D) h (detailed description of the cages in [55,56]). Only females capable of laying eggs during this period were used in the experiments.

The experimental units consisted of a detached bean leaf (4.0 × 4.0 cm) on water-saturated foam cubes in acrylic boxes (detailed description of the experimental units in [7]). The sex-determination units for the offspring generation were similarly constructed as the experimental units but were smaller (2.0 × 2.0 cm). To feed the predators during an experiment, spider mites were added to the experimental units. Eggs were reared under constant climatic conditions of 30 ± 2 °C, 60 ± 15% RH, and 16:8 (L:D) h in a walk-in climate chamber until the mites reached adulthood.

### 2.4. Reproduction of Predator and Prey under Heat Waves

The fertile females were singly transferred onto the experimental units, whereupon non-acclimated females were exposed to either mild or extreme heat waves (no correlation between juvenile and adult thermal environments) and acclimated females to heat wave conditions corresponding to their thermal environments during juvenile development. The predator females were provided with 150 spider mite eggs, which were renewed together with the bean leaf every third day. The prey females were provided with a fresh bean leaf on day 6. Thus, food availability was not a limiting factor for either species. For both species, the state of the individual females (alive, dead, or escaped) and the number of deposited eggs per female were recorded once every day over a period of 10 days. The same applied to the consumption rate of the predator females (number of prey eggs consumed per day). The feeding damage on the leaves caused by prey was assessed on days 5 and 10 by measuring the leaf area with feeding scars using a transparent mm^2^ plastic screen placed above the leaf surface. Thus, prey consumption was expressed as the amount of leaf area (in mm^2^) destroyed by a female averaged over a period of either 5 or 10 days. The experiments were conducted in the programmable Panasonic incubator MLR-352H-PE (humidity variation ±5% RH, temperature variation ±0.5 °C).

Every day, two eggs from each ovipositing female were randomly selected and placed singly on the sex-determination units to determine their volume and their subsequent gender, while the surplus eggs were removed after being counted. If a female produced one or two eggs during a day, all her eggs from that day were used in the analysis. A non-destructive method was applied to assess the volume of an egg by measuring (in µm) its semi-major axis (*a*) and semi-minor axis (*b*) (in the case of an ellipsoid predator egg) or its radius (*r*) (in the case of a globular prey egg), using a transmitted-microscope system, the Leica DMS 1000 (Leica Microsystems, Wetzlar, Germany), and the software Leica Application Suite X (LAS X 3.7.4.23463, Leica Microsystems, Wetzlar, Germany). Subsequently, the volume (in μm³) of an egg was calculated from either $V=\frac{4}{3}\pi ab^{2}$ or $V=\frac{4}{3}\pi r^{3}$, depending on species. After measuring their volumes, the eggs on the sex-determination units were reared to adulthood, allowing sex determination based on their sex-specific body size and shape (1927 of 15,213 prey eggs, 762 of 2221 predator eggs).

### 2.5. Reproduction of the Prey under Predation Risk and Heat Waves

**Figure 1:**
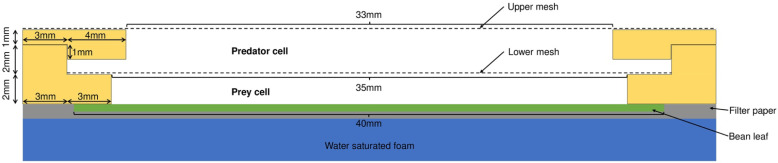
Schematic view of the experimental unit with the predator and prey cells.

Only predator-naïve (no experience with predator-related cues) and heat wave-exposed prey females were used in these experiments (i.e., heat wave conditions applied during adulthood were identical to those experienced during juvenile development). Prey females were exposed to the following cue treatments under either mild or extreme heat wave conditions: (1) no predation risk (control), (2) low predation risk (volatile predator cues), (3) high predation risk (contact predator cues), and (4) both low and high predation risk (volatile + contact cues). To arrange such scenarios, we modified the experimental set-up according to the templates used by [25]. A two-part acrylic quadratic frame was placed across the detached bean leaf (4.0 × 4.0 cm) with a single *T. urticae* female on a water-saturated foam cube in a plastic box. The cascaded inner parts of both frames interlocked the lower with the upper frame but also allowed the removal of the latter. The fine meshes (0.1 mm opening) were glued on the upper side of the upper frame and on the inner gradation of the lower frame. The entire closed frame on the bean leaf constituted two separate chambers: the prey cell between the leaf and the lower mesh, and the predator cell between the lower and upper mesh (Figure 1). This arrangement allowed for the placement of predator and prey inside their respective cells without physical contact between them, but at the same time enabled the prey to perceive predator-specific volatile cues.

Single, gravid prey females were placed on a bean leaf, and the two-part frame was placed across the leaf. Depending on the type of predation risk, the following procedures were applied: (i) control: the predator cell was left empty; (ii) low risk: two predator females and two prey females were placed in the predator cell; and (iii) high risk: two predator females and two prey females were placed on the bean leaf 24 h before an experiment started, while the predator cell was left empty. After 24 h, the predator females and their eggs (if any) were removed, and the dead spider mite females were left on the leaf. Surviving prey females were killed with a needle; (iv) combined risk: the female prey was exposed to both volatile cues from the predator cell and to contact cues in the prey cell. An experiment lasted for 48 h. The female survival and their number of deposited eggs were recorded every 24 h. Additionally, the leaf damage caused by spider mite feeding was measured with a transparent mm^2^ screen at the end of an experiment. For each combination of heat wave and predation risk treatment, 40 females were used (in total, 320 females). The experiments were conducted as a randomized block design consisting of four treatments per block using the Panasonic incubator MIR-154-PE (no humidity control, temperature variation ±0.5 °C). Relative humidity (RH) was recorded with data loggers (Testo 174 H), which corresponded approximately to the daily variations depicted in Table 1 (high RH values at low temperatures and vice versa).

### 2.6. Statistical Analyses

All statistical analyses were performed by means of SAS OnDemand [SAS Institute 2021, Cary, NC (USA)], except the analyses of acclimation- and heat wave-effects on escape and survival within and between the species based on the Breslow test. For this purpose, we used SPSS, version 27 [Armonk, NY (USA)].

#### 2.6.1. Survival and Escape Analyses

The likelihood that an individual is alive and present on a leaf disc at time *t* was modeled by means of Kaplan–Meier analyses. Lost individuals were either categorized as dead (when found on the leaf disc) or as having escaped. The number of surviving or remaining individuals at time *t* = 0 was used as the reference point for survival or escape probabilities, i.e., *f*(*t* = 0) = 1, where *f*(*t*) denotes the probability of being alive on a leaf disc at time *t*. Breslow tests for homogeneity of odds ratios were used to compare the differences between Kaplan–Meier functions within and between the species [57]. First, pooled over acclimation and heat waves, the total loss, escape, and survival functions were compared with respect to differences between the two species. Second, the acclimation and heat wave effects on the escape and survival functions were evaluated separately for predator and prey.

#### 2.6.2. Effects of Heat Waves and Female Age on Oviposition, Predation, and Egg Volume

First, the shifts in the oviposition rates induced by heat waves were compared with respect to differences between the two species, excluding the effects of acclimation and female age, by calculating the overall ratios between the average daily oviposition rates per female assessed at extreme and mild heat wave conditions. The ratios were obtained as *m = a/b*, where *a* and *b* denote the average oviposition rate of females exposed to extreme and mild heat wave conditions, respectively. The ratios were calculated using either censored data (females that were alive at the end of the experiments) or uncensored data (females that either died or escaped during the experiments were included in the data set). The 95% confidence limits for the ratios and the *p-*value for the difference between the two ratios were calculated according to Colquhoun [58]. The increase in oviposition rates of females exposed to extreme heat waves relative to those exposed to mild heat waves was assessed as 100∙(*a−b*)/*b*%. Second, we used the same generic function to model age-dependent oviposition rates, predation rates, and egg volumes, as follows:

(1) $$y\left(t\right)=at^{b}e^{ct+dt^{2}}\;,$$

where *t* is the age of a female, expressed as the time since the female eclosed, and *y*(*t*) is the predicted value of the dependent variable at age *t*, while *a, b, c,* and *d* are shape parameters. The underlying model ensures that the dependent variable cannot have negative values. Besides, if Equation (1) describes oviposition rates, *b >* 0 ensures that *y*(*t*) will be 0 at the time of enclosure. Equation (1) can be linearized by taking the natural logarithm of both sides of the equation, yielding the following:

(2) $$\ln y\left(t\right)=\beta_{0}+\beta_{1}\ln t+\beta_{2}t+\beta_{3}t^{2}+\varepsilon \;,$$

where *β*_0_ ≡ ln *a*, *β*_1_ ≡ *b, β*_2_ ≡ *c* and *β*_3_ ≡ *d. ε* denotes the residual error when Equation (2) is fitted to experimental data. *ε* is assumed to be normally distributed with a 0 mean and a variance that is independent of *t.* Equation (2) allows for the incorporation of additional independent variables, such as heat wave conditions during juvenile development and adulthood and the sex of the laid eggs. Since the females eclosed over a period of one day, their average age at the first assessment of *y*(*t*) was set to *t* = 3.5 days and to 12.5 days at the last assessment.

Equation (1) was fitted to data by means of a non-linear fitting procedure (PROC NLIN), while Equation (2) was analyzed by means of a repeated measures analysis using generalized estimating equations (PROC GEE). The statistical model used the unique ID code of each female as a random factor to account for the fact that the same females were followed over 10 consecutive days, thereby providing the significance of the between-subject factors (i.e., acclimation and heat wave treatment). When oviposition and consumption rates were analyzed, one was added to the observed values of *y*(*t*) prior to taking the logarithm.

The full models included all the main factors as well as their interactions. Non-significant terms (i.e., *p >* 0.05) were stepwise omitted from the model based on type III sum of squares (SS). Each species was analyzed separately, and only females that survived during the entire experimental period were used in the analysis.

#### 2.6.3. Effect of Heat Waves on the Feeding of *T. urticae*

Since feeding rates of *T. urticae* females were not assessed on a daily basis, only data for the average daily consumption rate per female over a period of 10 days (when mites were between 3.5 and 12.5 days old) were available for the analysis. Females that died during an experiment were excluded from the analysis. Data were analyzed by means of a two-way ANOVA (using PROC GLM), with heat wave conditions during adulthood and acclimation (yes, no), as well as their interactions, used as predictor variables in the full model. Non-significant terms were omitted from the model as described above.

#### 2.6.4. Effect of Heat Waves on the Sex Ratio of Eggs

The sex ratio of offspring produced by females exposed to different heat wave conditions was analyzed by means of logistic regression using PROC GENMOD. As the dependent variable, we used *n_f_/n*, where *n_f_* is the number of female eggs and *n* is the sum of female and male eggs produced by each female over a period of 10 days, while the predictor variables were heat conditions during egg laying and whether a female had been acclimated or not. The full model also included the interaction between the two factors. The dependent variable was assumed to be binomially distributed with a logit link function (i.e., dist. = binomial, link = logit). The residuals were scaled for over-dispersion to reduce the risk of type I errors. Each species was analyzed separately, and only females that survived the entire experimental period were used in the analyses. Non-significant terms were omitted from the model as described above.

#### 2.6.5. Effects of Heat Waves and Predation Risk on Feeding and Oviposition of *T. urticae*

The feeding rates of female prey exposed to predation risk as a potential stress factor were analyzed by a two-way ANOVA with heat wave conditions and predation risk as the independent variables and the leaf area damaged per female over a period of two days as the dependent variable. The analysis was conducted by means of PROC GENMOD, assuming that the dependent variable was normally distributed (*dist.* = normal; *link* = identity). The full model contained the main factors as well as their interactions. Non-significant terms were omitted from the reduced model, using type III sum of squares (SS) as a selection criterion. Post-hoc comparisons between all four levels of predation risk (control, low predation risk, high predation risk, and low + high predation risk) were carried out by means of Tukey’s test, while Dunnett’s test was applied to test whether the control group was significantly different from the three other groups. Oviposition rates were analyzed in the same way as feeding rates, except that the dependent variable (the number of eggs produced per female over a period of two days) was assumed to be Poisson distributed with a logarithmic link function. The pscale option was used to adjust for the potential effects of over- and under-dispersion in data.

## 3. Results

### 3.1. Heat Wave Effects on Survival and Escape Rates

#### 3.1.1. Species Comparisons

The total losses caused by escaped and dead individuals over the experimental period of 10 days differed between species (Kaplan–Meier analysis, Breslow test: $\chi_{1}^{2}$ = 57.720, *p* < 0.001). Thus, 40 out of 188 predator females (21.28%) and 108 out of 181 prey females (59.77%) remained alive on the detached bean leaves up to day 10, respectively. Pooled over heat waves and acclimation, the escape functions (cumulative residents regressed on escaping times), but not the survival functions (cumulative survivors regressed on residence times), were different between predator and prey. Hundred and thirty-four out of 188 (71.28%) predators and 20 out of 181 (11.05%) prey escaped from the leaves until day 10, respectively. Of the remaining females, 40 out of 54 (74.07%) predators and 108 out of 161 (67.08%) prey survived the experimental period of 10 days, respectively (Breslow tests, escape: $\chi_{1}^{2}$ = 129.799, *p* < 0.001; survival: $\chi_{1}^{2}$ = 1.685, *p* = 0.194).

#### 3.1.2. *Phytoseiulus persimilis*

**Figure 2:**
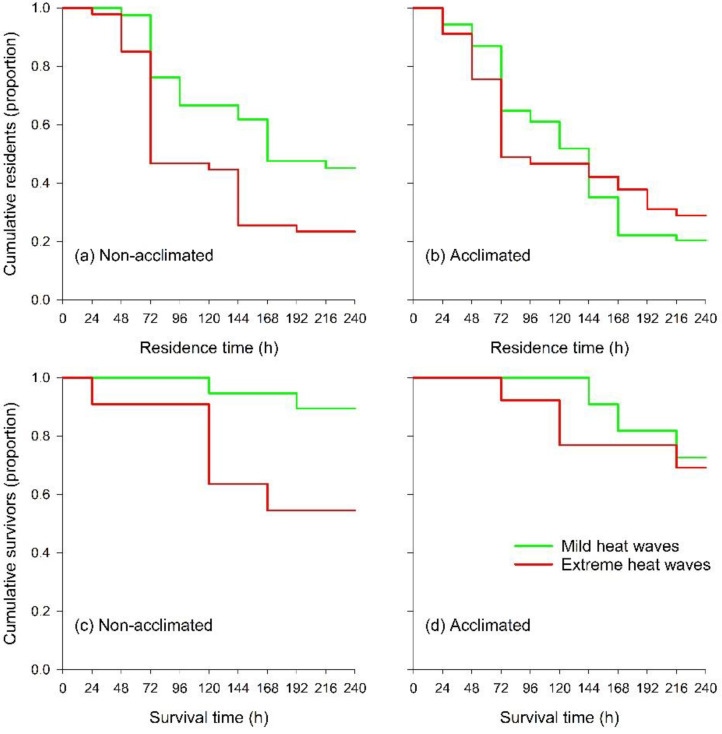
Escape functions (**a**,**b**) (cumulative mites staying on the arena plotted against residence times) and survival functions (**c**,**d**) (cumulative survivors plotted against survival times) of non-acclimated (**a**,**c**) and acclimated (**b**,**d**) *Phytoseiulus persimilis* females when exposed to mild 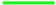 or extreme 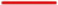 heat waves over 10 days.

Pooled over acclimation, the escape functions of the predator were affected by heat waves (Breslow test: $\chi_{1}^{2}$ = 6.409, *p* = 0.011). More females escaped during extreme heat waves (73.91% versus 68.75%), resulting also in lower residence times compared to mild heat waves (5.17 ± 0.33 SD days versus 6.17 ± 0.30 days). Pairwise comparisons, however, revealed that only non-acclimated females responded significantly differently to heat waves, as six out of 47 (12.77%) remained when heat waves were extreme, while 17 out of 42 (40.48%) remained after mild heat waves (Breslow test: $\chi_{1}^{2}$ = 10.10, *p* = 0.001) (Figure 2a,b). The corresponding values for acclimated females were nine out of 45 (20.00%) and eight out of 54 (14.81%) (Breslow test: $\chi_{1}^{2}$ = 0.381, *p* = 0.537).

Pooled over acclimation, the survival functions of the non-escaping predators were affected by heat waves: 25 out of 30 (83.33%) and 15 out of 24 (62.50%) females survived under mild and extreme heat waves, respectively (Breslow test: $\chi_{1}^{2}$ = 5.399, *p* = 0.020). Similarly, the acclimation status of the females influenced their survival functions. When exposed to extreme heat waves, six out of 11 (54.55%) non-acclimated females survived, whereas 17 out of 19 (89.47%) survived under mild heat waves (Breslow test: $\chi_{1}^{2}$ = 7.571, *p* = 0.006). The survival functions of the acclimated females were not affected by heat waves (mild versus extreme heat wave conditions: eight out of 11 (72.73%) survived versus nine out of 13 (69.23%), Breslow test: $\chi_{1}^{2}$ = 0.186, *p* = 0.666) (Figure 2c,d).

#### 3.1.3. *Tetranychus urticae*

**Figure 3:**
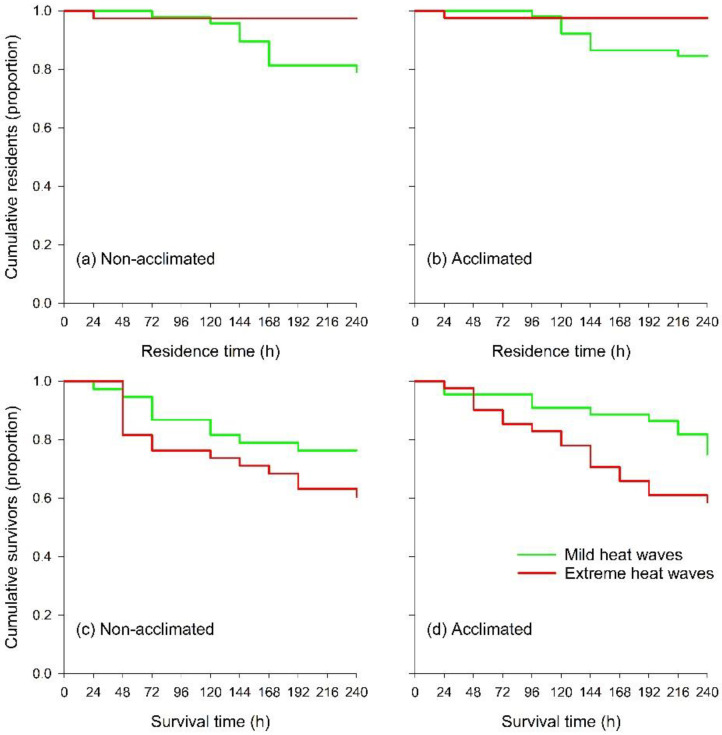
Escape functions (**a**,**b**) (cumulative residents plotted against residence times) and survival functions (**c**,**d**) (cumulative survivors plotted against survival times) of non-acclimated (**a**,**c**) and acclimated (**b**,**d**) *Tetranychus urticae* females when exposed to mild 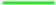 or extreme 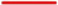 heat waves over 10 days.

Pooled over acclimation, the escape functions of the prey were significantly affected by heat waves (Breslow test: $\chi_{1}^{2}$ = 7.242, *p* = 0.007). Eighteen out of 100 (18.00%) females escaped during mild heat waves, compared with only two out of 81 (2.47%) during extreme heat waves. However, the acclimation status of the females affected their escape response to heat waves. Non-acclimated females had higher escape rates under mild than under extreme heat waves (10 out of 48 (20.83%) versus one out of 39 females (2.56%); Breslow test: $\chi_{1}^{2}$ = 4.330, *p* = 0.037). Acclimated females also tended to escape more frequently under mild heat waves [eight out of 52 (15.38%) versus one out of 42 females (2.38%)], but the difference was not significant (Breslow test: $\chi_{1}^{2}$ = 3.017, *p* = 0.082) (Figure 3a,b).

Pooled over acclimation, the survival functions of the prey were affected by heat waves (Breslow test: $\chi_{1}^{2}$ = 6.488, *p* = 0.011). Of the non-escaping females, more died under extreme heat waves [33 out of 79 (41.77%)] than under mild heat waves [20 out of 82 (24.39%)]. The survival functions of the non-acclimated females were not affected by heat waves [mild heat waves: nine out of 38 (23.68%) died; extreme heat waves: 15 out of 38 (39.47%) died; Breslow test: $\chi_{1}^{2}$ = 2.162, *p* = 0.141]. In contrast, more acclimated females died under extreme heat waves [mild heat waves: 11 out of 44 (25%); extreme heat waves: 18 out of 41 (43.90%)]; (Breslow test: $\chi_{1}^{2}$ = 4.368, *p* = 0.037). Females acclimated to mild heat waves had higher survival rates than non-acclimated females, whereas the two groups responded in the same way to extreme heat waves (Figure 3c,d).

### 3.2. Heat Wave Effects on Feeding Rates

#### 3.2.1. *Phytoseiulus persimilis*

**Figure 4:**
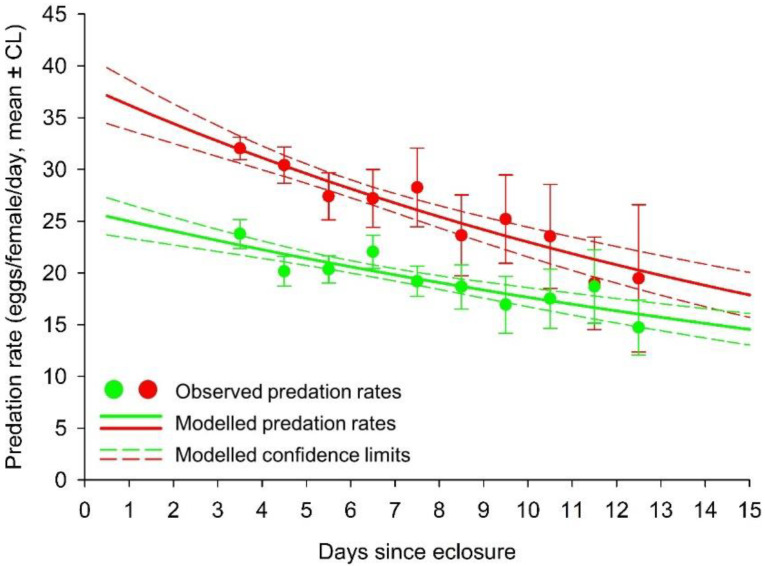
Heat wave effects (mild = 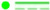, extreme = 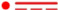) on the observed and modelled predation rates based on Equation 2a of the predator *P. persimilis* over time with 95% confidence limits (CL). The parameters were estimated as *a* = 38.0805 (1.5148 SE) and *c* = −0.0505 (0.0065) for extreme heat waves and *a* = 25.9780 (1.0046) and c = −0.0387 (0.0058) for mild heat waves.

Equation (1) explained 92.2% and 90.3% of the total variation in the observed predation rates for *P. persimilis* females exposed to extreme and mild heat waves, respectively. Since neither *b* nor *d* was significantly different from zero, *t^b^* was set to one and *dt*^2^ to zero, resulting in a model predicting that predation rates of *P. persimilis* females decline monotonously with age. The GEE analysis indicated that acclimation did not contribute to explaining predation rates, so this factor was excluded from the full model. Additionally, the interaction between heat waves and female age was non-significant ($\chi_{1}^{2}$ = 2.86; *p* = 0.091). The reduced model indicated that predation rates were significantly higher ($\chi_{1}^{2}$ = 27.85; *p* < 0.0001) at extreme heat wave conditions compared with mild conditions (27.92 ± 0.47 vs. 20.17 ± 0.31 eggs/female/day when averaged over 10 days) and that the predation rates decreased significantly with female age ($\chi_{1}^{2}$ = 30.15; *p* < 0.0001) (Figure 4).

#### 3.2.2. *Tetranychus urticae*

As with the predator, extreme heat waves stimulated feeding rates of *T. urticae* females (14.79 ± 0.96 vs. 11.70 ± 0.43 mm^2^/female/day) (*F*_1.105_ = 70.90; *p* < 0.0001). Acclimation also affected feeding of the prey, since non-acclimated females consumed significantly (*F*_1,105_ = 8.22; *p* = 0.0050) more than acclimated females (13.81 ± 0.79 vs. 12.32 ± 0.60 mm^2^/female/day). The interaction term between heat waves and acclimation was non-significant (*F*_1,105_ = 3.52; *p* = 0.0632).

### 3.3. Heat Wave Effects on Oviposition Rates

#### 3.3.1. Species Comparisons

Over a period of 10 days, female predators exposed to mild heat waves produced on average 11.09 ± 0.98 (*n =* 93) eggs/female and 13.84 ± 1.30 (*n =* 86) eggs/female when heat waves were extreme. The corresponding values for prey females were 75.02 ± 3.45 (*n =* 101) and 99.66 ± 7.42 eggs/female (*n =* 79).

Total egg production per female depended on whether the females died, escaped, or survived during the experimental period of 10 days. When acclimated and non-acclimated females were pooled, surviving predator females produced on average 21.20 ± 1.79 eggs/female (*n =* 25) when exposed to mild heat waves and 31.13 ± 3.53 eggs/female (*n =* 15) when exposed to extreme heat waves. The corresponding values for dead females were 12.40 ± 3.70 (*n =* 5) and 14.63 ± 3.68 (*n* = 8), and for escaped females, 6.97 ± 0.79 (*n =* 63) and 9.62 ± 0.94 (*n* = 63). Surviving prey females produced on average 96.75 ± 6.20 (*n =* 64) and 139.46 ± 6.20 eggs/female (*n =* 46) when exposed to mild and extreme heat waves, respectively. The corresponding values for dead females were 27.29 ± 6.48 (*n =* 17) and 37.00 ± 7.40 (*n =* 33). None of the spider mites escaped when heat wave conditions were extreme, while the average oviposition of escaping females under mild conditions was 46.05 ± 4.03 (*n =* 20) eggs/female.

Converted to daily oviposition rates, censored *P. persimilis* females exposed to extreme (*n* = 15) and mild heat waves (*n* = 25) produced 3.11 ± 0.274 and 2.12 ± 0.180 eggs/female/day, respectively, yielding a ratio of 1.469 ± 0.179. The corresponding values for *T. urticae* were 13.957 ± 0.619 eggs/female/day (*n =* 46) and 9.824 ± 0.183 (*n* = 62), yielding a ratio of 1.421 ± 0.068. The difference between the two ratios was insignificant (*t*_154_ = 0.250; *p =* 0.803). In contrast, the ratios were significantly different (*t*_362_ = 3.473; *p =* 0.0006) when based on all females. Thus, the average oviposition rates of uncensored *P. persimilis* females exposed to extreme and mild heat waves were 3.199 ± 0.115 (*n =* 93) and 1.788 ± 0.111 (*n =* 86) eggs/female/day, respectively, yielding a ratio of 1.789 ± 0.128. The corresponding values for *T. urticae* were 10.889 ± 0.667 (*n =* 79) and 8.696 ± 0.275 (*n =* 101) eggs/female/day, yielding a ratio of 1.252 ± 0.086. Including all females, a change from mild to extreme heat wave conditions had a much stronger relative impact on the predator than on the prey, as the former increased its oviposition rates by 78.85% compared with 25.22% for the prey.

#### 3.3.2. Species-Specific Heat Wave Effects on Oviposition Rates

The models based on Equation (1) provided a good fit to the oviposition rates of both species. Only the parameters *a*, *b*, and *c* were found to contribute significantly to the model, so *d* was set to zero. The model, which corresponds to the gamma distribution, explained 80.9% of the variation in the observed oviposition rates of *P. persimilis* when exposed to extreme heat waves and 73.4% when exposed to mild heat waves. The corresponding values for *T. urticae* were 81.3% and 89.1%, respectively.

##### *Phytoseiulus* *persimilis*

**Figure 5:**
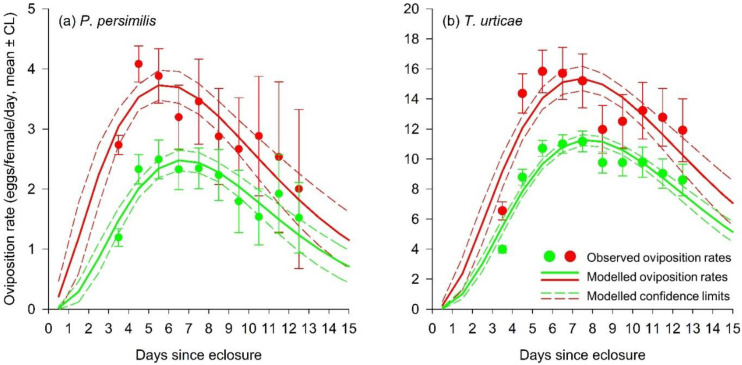
Effects of heat wave conditions (mild = 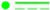 or extreme heat waves = 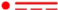) and days since enclosure on daily per capita oviposition rates of *P. persimilis* (**a**) and *T. urticae* (**b**) with 95% confidence limits (CL). For *P. persimilis,* the parameters of Equation (1) were estimated as *a* = 0.8970 (0.2893), *b* = 1.8765 (0.4005), and *c* = −0.3223 (0.0643) for extreme heat waves and *a* = 0.1683 (0.0651), *b* = 2.9610 (0.4409), and c = −0.4390 (0.0665) for mild heat waves. The parameters for *T. urticae* were estimated as *a* = 1.4600 (0.4371), *b* = 2.3705 (0.3158), and *c* = −0.3230 (0.0441) for extreme heat waves and *a* = 0.5416 (0.1124), *b* = 2.8919 (0.2117), and *c* = −0.3723 (0.0288) for mild heat waves.

The GEE analysis, based on Equation (2), revealed that acclimation effects on the number of deposited predator eggs were insignificant and were therefore omitted from the model. The same applied to the interaction terms between heat waves and female age, so the reduced model contained only three independent variables that were significant, namely heat wave conditions ($\chi_{1}^{2}$= 35.62; *p* < 0.0001), ln(*t*) ($\chi_{1}^{2}$= 29.43; *p* < 0.0001), and *t* ($\chi_{1}^{2}$= 30.58; *p* < 0.0001). Extreme heat waves increased average oviposition rates compared to mild heat waves [average eggs/female/day (±SE): 3.22 ± 0.09 vs. 2.01 ± 0.06]. Besides, oviposition rates peaked earlier when predator females were exposed to extreme heat waves (Figure 5a).

##### *T.* *urticae*

In concert with the predator, neither acclimation nor the interactions between heat waves and female age affected oviposition rates of the prey females. The reduced model showed that heat wave effects ($\chi_{1}^{2}$ = 19.66; *p* < 0.0001), ln(*t*) ($\chi_{1}^{2}$ = 106.47; *p* < 0.0001), and *t* ($\chi_{1}^{2}$ = 96.10; *p* < 0.0001) were highly significant. Extreme heat waves increased the average oviposition rates compared to mild heat waves (12.90 ± 0.27 versus 9.22 ± 0.13 eggs/female/day). Oviposition rate peaked around 7 days after enclosure, irrespective of heat wave conditions (Figure 5b).

### 3.4. Effects of Heat Waves and Maternal Age on Offspring Egg Size

When Equation (1) was fitted to the egg sizes of *P. persimilis* and *T. urticae*, it was found that *b* did not contribute significantly to the model, so the term *t^b^* was replaced by one. The reduced model explained very well the variation in the observed egg sizes, as reflected by *R*^2^ values close to one.

#### 3.4.1. *Phytoseiulus persimilis*

**Figure 6:**
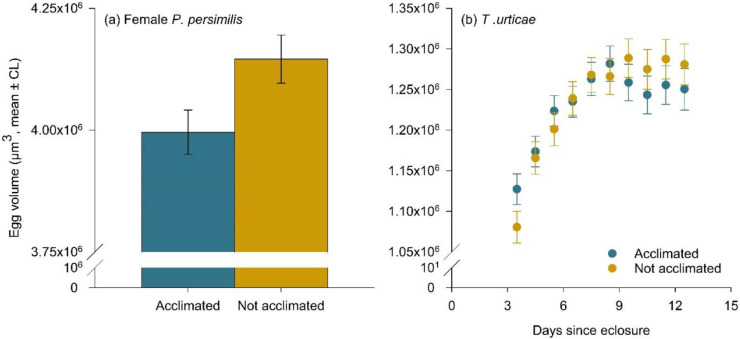
Effects of acclimation (acclimated = 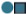 or not acclimated = 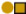) on egg sizes of female *P. persimilis* eggs (**a**) and a combination of effects of acclimation and days since enclosure on egg sizes of *T. urticae* pooled over the sex of eggs (**b**) with 95% confidence limits (CL).

**Figure 7:**
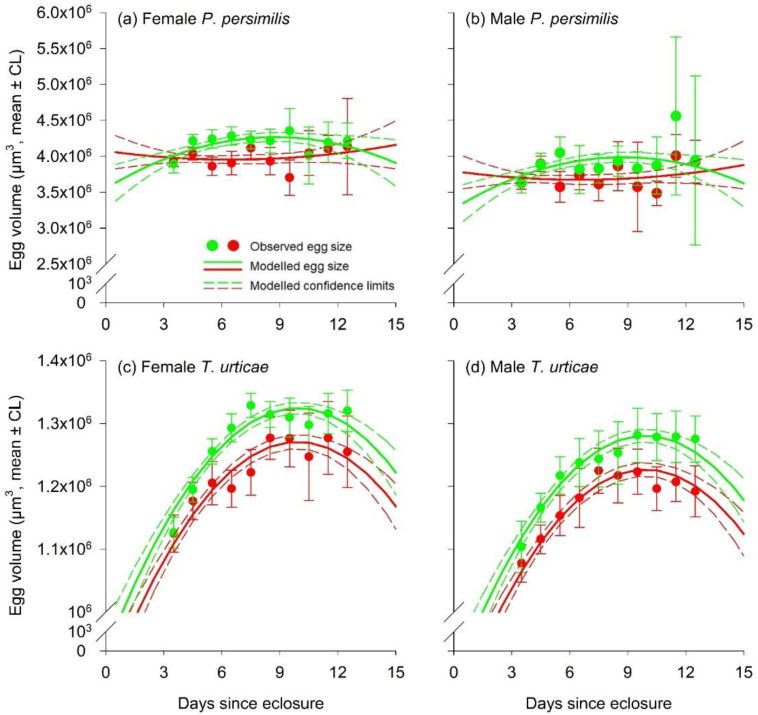
Effects of maternal age and heat wave conditions (mild = 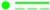 or extreme heat waves = 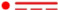) on volumes of female (**a**,**c**) and male (**b**,**d**) eggs of *P. persimilis* (**a**,**b**) and *T. urticae* (**c**,**d**) with 95% confidence limits (CL). Data for acclimated and non-acclimated females were pooled. For *P. persimilis,* the parameters of Equation 1 were estimated for female eggs laid by females exposed to extreme heat waves: *a =* 4.0776 (0.1523), *c* = −0.0116 (0.0124), and *d =* 0.00095 (0.00091) (*R*^2^ = 0.992); female eggs laid by females exposed to mild heat waves: a *=* 3.6088 (0.1406), *c* = 0.0436 (0.0121), and *d =* −0.00028 (0.00085) (*R*^2^ = 0.992); male eggs laid by females exposed to extreme heat waves: *a =* 3.7318 (0.1816), *c* = −0.0334 (0.0168), and *d =* 0.00033 (0.00012) (*R*^2^ = 0.989); male eggs laid by females exposed to mild heat waves: *a =* 3.5284 (0.2198), *c* = 0.0220 (0.0204), and *d =* −0.0010 (0.0015) (*R*^2^ = 0.989). For *T. urticae,* the estimated parameters for female eggs laid by females exposed to extreme heat waves: *a =* 1.0148 (0.0352), *c* = 0.0425 (0.0105), and *d =* −0.0020 (0.00072) (*R*^2^ = 0.991); female eggs laid by females exposed to mild heat waves: *a =* 0.9490 (0.0225), *c* = 0.0747 (0.0068), and *d =* −0.0044 (0.00045) (*R*^2^ = 0.993); male eggs laid by females exposed to extreme heat waves: *a =* 0.9194 (0.0309), *c* = 0.0633 (0.0099), and *d =* −0.0035 (0.00066) (*R*^2^ = 0.992); Male eggs laid by females exposed to mild heat waves: *a =* 0.9722 (0.0345), *c* = 0.0559 (0.0103), and *d =* −0.0028 (0.00067) (*R*^2^ = 0.990). Note that the predicted values have to be multiplied by 10^6^ to give egg volumes in μm^3^.

The most important factor affecting predator egg size was their sex, since female eggs were significantly larger than male eggs (+3.51%) ($\chi_{1}^{2}$ = 45.90; *p* < 0.0001). Female age (both as *t* and *t*^2^) affected egg size (*t*: $\chi_{1}^{2}$ = 6.09; *p* = 0.0136; *t*^2^: $\chi_{1}^{2}$ = 4.68; *p* = 0.0305). Heat wave conditions also affected egg size, as extreme heat wave conditions reduced egg size ($\chi_{1}^{2}$ = 7.42; *p* = 0.0065). However, the significant interaction terms of heat waves with *t* ($\chi_{1}^{2}$ = 9.28; *p* = 0.0023) and *t*^2^ ($\chi_{1}^{2}$ = 8.12; *p* = 0.0044) indicate that the heat wave effects varied with female age, but only under mild heat wave conditions. Thus, female predators of intermediate age laid larger eggs compared with both younger and older females. There was no overall effect of acclimation, but a significant interaction term between acclimation and sex reveals that male and female eggs were differently affected by acclimation. Thus, female eggs produced by acclimated mothers were significantly smaller than female eggs laid by non-acclimated mothers ($\chi_{1}^{2}$ = 5.40; *p* = 0.0201; see Figure 6a). However, since the effect of acclimation was rather small, acclimated and non-acclimated females were lumped together in Figure 7a,b.

#### 3.4.2. *Tetranychus urticae*

Female spider mite eggs were significantly larger than male eggs (+6.26%) ($\chi_{1}^{2}$ = 49.71; *p* < 0.0001). Females exposed to extreme heat waves produced significantly smaller eggs ($\chi_{1}^{2}$ = 23.64; *p* < 0.0001) than those experiencing mild conditions. Acclimation had a positive effect on egg sizes ($\chi_{1}^{2}$ = 10.43; *p* = 0.0012), but the effect of acclimation declined with age since the interaction term between acclimation and *t* was significant ($\chi_{1}^{2}$ = 13.82; *p* = 0.0002; see Figure 6b). Finally, female age significantly affected egg size (*t*: $\chi_{1}^{2}$ = 72.96; *p* < 0.0001; *t*^2^: $\chi_{1}^{2}$ = 58.27; *p* < 0.0001). The largest eggs were produced by older females (Figure 7c,d).

### 3.5. Heat Wave Effects on Sex Ratios

#### 3.5.1. *Phytoseiulus persimilis*

Of the 2221 predator eggs produced over a period of 10 days, 762 eggs (34.3%) laid by 137 females were sexed, and 496 (65.1%) of these eggs were female. Neither acclimation nor the interaction between heat waves and acclimation significantly contributed to the model. However, the proportion of female eggs was significantly lower (*F_1,135_* = 4.89; *p* = 0.0287) when their mothers were exposed to extreme heat waves (61.8% [95% CL: 57.2–66.2%]) compared with mild heat waves (69.2% [95% CL: 64.3–73.8%]).

#### 3.5.2. *Tetranychus urticae*

Of the 15213 prey eggs produced over a period of 10 days, 1927 eggs (12.7%) laid by 156 females were sexed, and 1126 (58.4%) of these eggs were female. Neither acclimation nor the interaction between heat waves and acclimation significantly contributed to the model, but heat waves (*F*_1,154_ = 8.84; *p* = 0.0034) did. The proportion of female eggs was significantly lower when the mothers were exposed to extreme heat waves (50.6% [95% CL: 44.1–57.2%]) compared with mild heat waves (63.1% [95% CL: 51.8–67.8%]).

### 3.6. Reproductive Performance of Prey under Heat Waves and Predation Risk

#### 3.6.1. Feeding Rates

**Figure 8:**
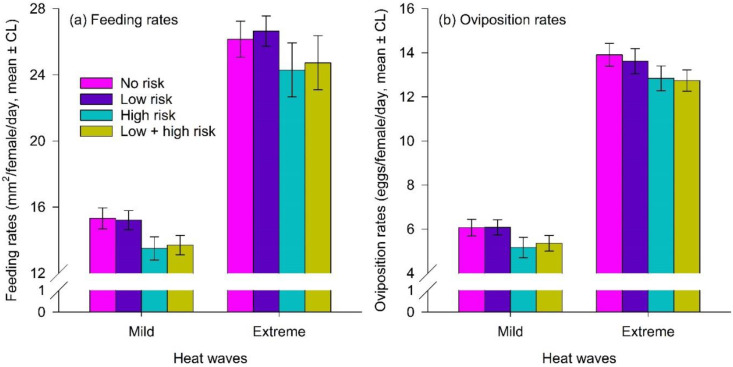
Effects of heat waves (mild or extreme) and predation risk [control 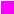
(no predator cues); low risk 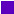
(volatile cues only); high risk 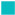
(contact cues only); low + high risk 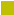
(combination of contact and volatile cues)] on the feeding (**a**) and oviposition rates (**b**) of *T. urticae* with 95% confidence limits (CL).

The analysis revealed that both heat waves and predation risk contributed significantly to the model, whereas their interaction was not significant (heat waves: *F*_1,300_ = 866.32; *p* < 0.0001; predation risk: *F*_3,300_ = 7.65; *p* < 0.0001; heat waves*predation risk: *F*_3,300_ = 0.16; *p* = 0.9259) (Figure 8a). Pooled over predation risk, females exposed to extreme heat wave conditions caused significantly more damage than those exposed to mild conditions (25.45 ± 0.35 vs. 14.43 ± 0.17 mm^2^/day; *p* < 0.0001). Pooled over heat waves, Tukey’s test revealed that the feeding damages of females without predation risk and females exposed to low predation risk were not different (20.81 ± 0.70 vs. 20.85 ± 0.71 mm^2^/day; *p_adj_* = 0.9842), but significantly higher than for females exposed to high predation risk (18.82 ± 0.76; *p_adj_* = 0.0029) and the combination of low and high predation risk (19.29 ± 0.77; *p_adj_* = 0.0213). Feeding damage in females exposed to high predation risk was not significantly different from females exposed to both risks (*p_adj_* = 0.9312) (Figure 8a).

#### 3.6.2. Oviposition Rates

Similarly, the analysis revealed that both heat waves (*F*_1,300_ = 2059.04; *p* < 0.0001) and predation risk (*F*_3,300_ = 10.24; *p* < 0.0001) significantly contributed to the model, whereas their interaction was not significant (*F*_3,300_ = 1.32; *p* = 0.2690) (Figure 8b). Pooled over predation risk, females deposited significantly more eggs under extreme heat waves than under mild heat waves (13.28 ± 0.14 vs. 5.67 ± 0.10; *p* < 0.0001), which corresponds to a reproduction gain of 134.22%. Pooled over heat waves, females without predation risk laid significantly more eggs (10.04 ± 0.48) than females exposed to high predation risk (8.95 ± 0.48) and to both low and high predation risk (9.10 ± 0.45) (*p_adj_ ≤* 0.0049 in all pairwise tests adjusted for multiple comparisons), whereas the differences in reproduction between females without predation risk (10.04 ± 0.48) and females exposed to low predation risk (9.80 ± 0.46), and between females exposed to high predation risk (8.95 ± 0.48) and females exposed both to low and high predation risk (9.10 ± 0.45) were insignificant (*p_adj_* ≥ 0.9451 in all pairwise tests adjusted for multiple comparisons). The lowering of reproduction induced by high predation risk or the combination of low and high predation risk ranged from 7.69% to 12.18% (Figure 8b).

## 4. Discussion

Our study provides experimental evidence for the complex interplay of species-, sex-, and trait-specific juvenile and adult thermal environmental effects on the reproductive performance of the predator *P. persimilis* and its prey *T. urticae*. Juvenile acclimation (identical juvenile and adult heat wave conditions) influenced escape behavior, mortality, and offspring egg sizes, whereas changes in oviposition and offspring sex ratios were induced only by the adult thermal environment. Additionally, predation risk triggered threat-sensitive reductions in the fecundity of prey, but these changes induced by predation risk were small compared to heat wave effects.

### 4.1. Escape Behavior and Survival under Heat Waves

In general, the total losses during the ten-day heat wave periods were much higher for predatory females than for prey females. This was mainly caused by the predator’s higher tendency to escape adverse environmental conditions, whereas differential mortality played a minor role. These species-specific responses are in line with the predictions of the trophic sensitivity hypothesis. Predators should be more sensitive to heat waves than prey due to their higher mobility, allowing them to move to thermally suitable habitats. In contrast, prey with limited mobility are bound to remain in heat wave-exposed habitats, evolving higher tolerance to heat waves through physiological adaptations [11]. Generally, these assumptions are supported by empirical evidence. Unless the food resources become overexploited or population densities are too high, spider mites tend to stay in the colony once it is established [59,60]. In contrast, as an active prey-searching predator, *P. persimilis* is highly mobile [61].

In detail, juvenile acclimation effects on escape behavior and mortality were species-specific. Non-acclimated predators responded strongly to extreme heat waves by increasing mortality and escape rates, whereas acclimated females were insensitive to heat wave conditions. To emigrate from a prey patch is a risky undertaking for the predator, because *P. persimilis* is specialized to prey on patchily distributed spider mites [48]. Consequently, juvenile acclimation effects in relation to escaping and mortality are likely to be adaptive. In contrast to the predators, prey females escaped more frequently under mild heat waves, which could be attributed to the lower daily minimum temperature limits (16 °C) under mild heat waves. Such low temperatures are unfavorable for adult females and reduce their fecundity [62,63] and lifespan [64]. On the other hand, emigration from a suitable food patch is also a suboptimal strategy as it involves the risk of encountering a predator. However, juvenile acclimation decreased this trend. In concert with the predator, extreme heat waves increased the mortality rates, but to a lesser extent in non-acclimated prey. Thus, juvenile acclimation seems to have benefits (fewer escaper) but also costs (higher mortality) for the prey. Finally, it cannot be excluded that genotypic differences among individuals also played a role because of the high total losses caused by heat wave-sensitive mortality and escape rates. Consequently, genotypic adaptation, phenotypic plasticity, or a mix of both mechanisms could have contributed to the observed changes in feeding, egg number and size, and sex ratios of predators and prey when exposed to mild and extreme heat waves.

### 4.2. Reproduction under Heat Waves

Increased feeding at higher temperatures is a common phenomenon in ectothermic predators [65] and herbivorous prey [66]. Our study organisms responded in a similar manner, which is in agreement with the Universal Temperature Dependence (UTD) of metabolism [67,68]. Theoretically, females can invest this surplus energy in maintenance demands (to compensate for increasing metabolic costs), reproduction (by increasing egg numbers), or offspring fitness (by increasing egg size). Because energy is limited, this usually leads to a trade-off between egg number and egg size, resulting in many, but small eggs, as in our study organisms or few large eggs [69,70,71,72]. Egg size is a significant evolutionary and ecological trait, influencing both maternal and offspring fitness, because investment in egg size is linked with decreased reproductive output and vice versa [71]. Such trade-offs are expected when (1) maternal food resources are limited [73], (2) offspring fitness is exclusively dependent on maternal investment (egg size), and (3) maternal body size results in morphological constraints [71]. First, maternal food stress can be excluded because both species were provided with ample food resources. Second, small egg sizes can have verifiable negative effects on offspring fitness [72], as demonstrated for both species when developing from small eggs [32,47]. However, extrinsic factors, such as the ambient temperature or food availability, may override the influence of intrinsic factors on offspring fitness under natural conditions [71]. Third, maternal body size may have influenced the egg sizes deposited by the predator but not the prey. Thermal developmental plasticity led to smaller adult female size in the predator under extreme heat waves [7], so that these maternal morphological and/or physiological constraints could be the reason for small eggs. Moreover, independently of heat waves, juvenile acclimation affected egg sizes of both species but in an opposite direction: prey eggs became larger, while female predator eggs became smaller. A potential cause of the sex-specific effects in the predator could be that the morphological traits of males, including egg size, are less sensitive to environmental stress than female traits. For example, the male body size of *P. persimilis* was found to be insensitive to food shortages and heat stress [8,74]. Therefore, modification of female egg size is more likely because of their higher plasticity compared to male eggs. The larger eggs deposited by the acclimated prey females are remarkable, since such mothers also exhibited lower consumption rates compared to non-acclimated females. The most parsimonious reason for this finding is that acclimated females have a higher energy efficiency than non-acclimated females, allowing them to produce larger eggs [75].

Additionally, both species modified their offspring sex ratios under heat waves in the same direction by producing relatively fewer female eggs, which resulted in slightly female-biased sex ratios in the predator and equally balanced sex ratios in the prey. Females of both mite species are able to manipulate the gender of their offspring, thereby lowering the female ratio under adverse environmental conditions, such as high population densities, food shortages, and high temperatures [38,39,73]. Proximately, reduction in female egg size may save energy because production of large female eggs is costly for heat-stressed predator females [32,73,76]. On the other hand, a lower female ratio is also associated with reduced fitness at the population level since it negatively affects the intrinsic rate of increase *r_m_*, causing a decline in population growth [77].

### 4.3. Reproductive Performance of Prey under Predation Risk and Heat Waves

In agreement with previous studies dealing with the same predator–prey couple [25,26,78,79,80], the prey responded to the presence of contact cues, indicating a high predation risk, by reducing feeding activity and oviposition. These findings can be interpreted as a trade-off between the acquisition of food resources and the avoidance of predation [81]. The potential physical presence of a predator may require increased attention from the prey at the expense of feeding activities and, accordingly, reduce reproduction. However, this conflict between feeding, reproduction, and anti-predator behavior was not evident in prey exposed to volatile cues (indicating low predation risk). These findings may provide an example of a threat-sensitive anti-predator response: investment in anti-predator behavior under high risk and investment in reproduction under low risk. Such fine-tuned prey responses imply the accurate perception and differentiation of predator-induced cues. In a similar experimental set-up, prey lowered reproduction when exposed to volatile predator cues at constant temperatures of 26 °C [25]. The discrepancy between the two studies raises the question of whether our fluctuating thermal conditions with high temperature peaks have shortened the persistence of volatile cues and/or the ability of the prey to perceive them correctly. On the other hand, the focal prey in our experiments was permanently exposed to volatile cues, which differed in their origin: directly from the predators and indirectly from attacked and killed prey. Such multiple predator-induced signals should markedly decrease perception errors in the prey [82]. Accordingly, we argue that prey correctly perceived the predator’s cues and responded in a risk-sensitive manner.

Finally, the positive influence of heat waves on reproduction (+134.11% under extreme heat waves) more than outweighed the negative effects of high predation risk, which marginally lowered egg production of the prey (−10.11%). Consequently, the capacity for population increase of the prey *T. urticae* should not be strongly limited under extreme heat waves despite a high predation risk.

### 4.4. Possible Consequences for Biological Control

Irrespective of species, females laid more but smaller eggs during extreme heat waves. However, juvenile acclimation dampened this effect in spider mite eggs, while the opposite applied to female eggs produced by the predator. These findings may affect the likelihood of successful biological control during heat waves because female egg size and size at adulthood are positively correlated in the predator [83]. Small females may produce fewer eggs and suffer from higher mortality than standard-sized females [47]. However, even more important for achieving control of spider mites during heat waves is the high loss of predators due to emigration, as about two-thirds of the females escaped from the leaves during the experimental period of ten days. Especially young females exposed to extreme heat waves showed a high tendency to emigrate. However, the higher relative reproductive output of the predator under extreme heat waves compared to prey was mainly attributed to these young, escaping predator females (Figure 5a). Therefore, although both species were capable of increasing their reproductive output during extreme heat waves, it seems to be the prey that will gain the greatest advantage from rising temperatures, at least at the local scale. For biological control in crop systems, the key question is whether a predator leaving a prey patch will survive until it succeeds in finding a suitable prey patch, which is a prerequisite for long-term biocontrol based on metapopulation dynamics [84]. Under lab conditions of constant temperatures and photoperiod*, P. persimilis* exhibits a diel predation pattern that seems to be affected by daytime: In summer, feeding activities are reduced around midday and reach their maximum in the late afternoon [85]. It needs further investigations to evaluate whether such feeding behavior is inherently coupled with the need to avoid high temperatures, which usually occur around noon, and to return to the prey patches in the afternoon when the climatic conditions here are more comfortable again. In such a scenario, biocontrol of spider mites by the predator *P. persimilis* may also work during heat waves. Another aspect to be considered is the origin of the predator populations, as this may affect their heat resistance through local adaptation. For example, field-collected *P. persimilis* populations were able to suppress spider mites in citrus orchards under heat waves, whereas another predatory mite, *Neoseiulus californicus*, originating from a commercial producer reared under optimal conditions [86], was not. The predators used in our study also came from a commercial producer and might be less heat-resistant than naturally occurring populations. However, while field populations of the predator *P. persimilis* and other predatory mites are usually established in permanent crop systems, such as orchards and vineyards, this is not the case in seasonal crops. Here, growers frequently depend on enemies delivered by commercial producers to cope with spider mite outbreaks.

## 5. Conclusions

Our results indicate that the thermal history of this predator–prey couple affected their adult life history traits during heat waves. The interaction of the juvenile and adult thermal environments of predator and prey changed the survival probabilities, escape rates, and egg sizes, whereas fecundity and offspring sex ratios were only influenced by the adult thermal environment. At the individual level, coping with heat waves seems to have higher costs for the predator than for the prey in relation to parental and offspring juvenile development [7] and reproduction. However, other factors (e.g., plant systems, management methods, and microclimate), which were excluded from our lab studies, may affect the population dynamics of predators and prey in more complex natural settings. Our results cannot be extrapolated to the practice of spider mite control without taking system-specific factors into consideration. Consequently, we intend to conduct population experiments in a tri-trophic system consisting of a crop, the herbivorous spider mite, and its predator in order to evaluate to what extent heat wave conditions affect biological control at the plant scale.

## Figures and Tables

**Table 1:** Diurnal variations of temperature and relative humidity (RH) mimic mild and extreme heat waves at long-day conditions (L:D = 16:8 h).

Time of the Day	Mild Heat Waves		Extreme Heat Waves	
	Temperature (°C)	RH (%)	Temperature (°C)	RH (%)
00:00–02:00	18.0	75.0	24.0	65.0
02:00–04:00	17.0	80.0	23.0	70.0
04:00–06:00	16.0	85.0	22.0	75.0
06:00–08:00	19.0	75.0	25.0	65.0
08:00–10:00	27.0	60.0	33.0	50.0
10:00–12:00	30.0	50.0	36.0	50.0
12:00–14:00	32.0	50.0	38.0	50.0
14:00–16:00	29.0	55.0	35.0	50.0
16:00–18:00	24.0	65.0	30.0	55.0
18:00–20:00	22.0	70.0	28.0	60.0
20:00–22:00	19.0	75.0	25.0	65.0
22:00–00:00	18.0	75.0	24.0	65.0
MEAN	22.6	67.9	28.6	60.0

## Data Availability

The data that support the findings of this study are openly available in Zenodo at https://doi.org/10.5281/zenodo.7554734 (accessed on 27 January 2023).
